# MoS_2_ Nanosheet/ZnO Nanowire-Functionalized Optical Fiber LSPR Biosensor for Sensitive Detection of 2,4-D Herbicide Residues

**DOI:** 10.3390/nano16130829

**Published:** 2026-07-06

**Authors:** Huibo Han, Shuai Wang, Rui Min, Ragini Singh, Bingyuan Zhang, Santosh Kumar

**Affiliations:** 1 Shandong Key Laboratory of Optical Communication Science and Technology, School of Physics Science and Information Technology, Liaocheng University, Liaocheng 252059, China; 2 Bay Area School of Applied Psychological Sciences, Beijing Normal University, Zhuhai 519087, China; 3 Faculty of Psychology, Beijing Normal University, Beijing 100875, China; 4 Department of Biotechnology, Koneru Lakshmaiah Education Foundation, Vaddeswaram 522302, Andhra Pradesh, India; 5 Centre of Excellence for Nanotechnology, Department of Electronics and Communication Engineering, Koneru Lakshmaiah Education Foundation, Vaddeswaram 522302, Andhra Pradesh, India

**Keywords:** optical fiber biosensor, localized surface plasmon resonance (LSPR), 2,4-Dichlorophenoxyacetic acid (2,4-D), MoS_2_ nanosheets, ZnO nanowires, environmental monitoring

## Abstract

2,4-Dichlorophenoxyacetic acid (2,4-D) is an extensively applied organic compound, primarily for agricultural weed control and plant growth agents. Although 2,4-D usually exists in the environment in low volumes, the detection of 2,4-D is critical for human health and environmental safety. In this work, a biophotonic biosensor was fabricated by coating the surface of a tapered optical fiber with gold nanoparticles (AuNPs) to excite the localized surface plasmon resonance (LSPR) and functionalizing the fiber with molybdenum disulfide nanosheets (MoS_2_-NSs)/zinc oxide nanowires (ZnO-NWs) to extend the effective sensing area. Due to the inhibitory effect of 2,4-D on the hydrolytic activity of ALP, the refractive index (RI) around the sensor surface changes, leading to a shift in the LSPR peak wavelength. According to this sensing technique, the sensor can detect concentrations in the range of 1–10 mg/L, with a limit of detection (LOD) of 0.29 mg/L. The stability, repeatability and selectivity tests show that the sensor has good stability and selectivity. In the actual sample detection experiment, the recovery rates of apples and Chinese cabbage were 96.2–100.4% and 83.8–108.8%, respectively, which indicated that the detection method had good accuracy for the detection of target substances in actual samples. Thus, the proposed sensor has an important application in the detection of 2,4-D herbicides.

## 1. Introduction

2,4-Dichlorophenoxyacetic acid (2,4-D) has been commercialized since World War II and is mainly used as an agricultural herbicide and plant growth agent due to its low dosage and low cost [1]. According to surveys, people have detected excessive levels of 2,4-D in crops such as pepper, bean sprouts, and apples [2]. 2,4-D promotes cell division and elongation at low concentrations and extends the shelf life of fruits [3]. 2,4-D is found in soil and water resources because it is easy to dissolve in water, has a low soil adsorption coefficient, and has strong mobility [4]. Utilized in over 1500 items, 2,4-D presents significant environmental risks, such as the possibility of cancer and interference with the hormonal and central neurological systems of living things [5]. At doses of 50 mg/kg or more of 2,4-D, the renal transport mechanisms are rapidly saturated [6]. Although concentrations of 2,4-D residues in the environment are usually low, chronic exposure to 2,4-D herbicide residues may increase the risk of cancer, disrupt endocrine hormone balance in the body, and adversely affect normal metabolic and detoxification functions of the liver [7]. Therefore, their detection is essential for health and environmental safety [8]. Detailed studies on the adsorption mechanism of 2,4-D by Samanth et al. [9], Vinayagam et al. [10], and Sridevi et al. [11]. While these studies have not well addressed the long-term effects of the complex components in the water matrix on the adsorption effectiveness, they have important implications on water purification and environmental remediation. Alkaline phosphatase (ALP) is not only an important member of the phosphatase subfamily but also a widely distributed phosphate monoester hydrolase in the human body [12]. Since 2,4-D plays an inhibitory role in the hydrolysis reaction between ALP and disodium benzene phosphate, the inhibition data of the hydrolysis reaction between ALP and disodium benzene phosphate by 2,4-D can be used to determine whether 2,4-D herbicide residues are present [13].

Recently, Botre et al. proposed and discussed an electrochemical biosensor for dual inhibition of enzymes, which depends on the combination and catalytic function of ALP and glucose oxidase and is mainly used for the detection of 2,4-D. However, the sensor has a small detection range [14]. Similarly, Bollella et al. developed an electrochemical biosensor that characterizes the dynamic interaction between ALP and 2,4-D as a representative inhibitor, ultimately measuring 2,4-D by measuring data on ALP inhibition. However, this sensor has a relatively low sensitivity [15]. Therefore, the development of a novel biophotonic biosensor for 2,4-D is particularly urgent. The biophotonic biosensor is a flexible optical fiber sensor developed to effectively induce the localized surface plasmon resonance (LSPR) effect through the amplification of the evanescent wave [16]. It leverages the characteristics of plasmon waves and the biophotonic structure of optical fibers to detect biological analytes [17]. Furthermore, the biophotonic fiber configuration confers numerous advantages for biosensing applications [18]. It offers an increased surface area for modification, enabling the secure binding of nanomaterials, enzymes, or antibodies, thereby boosting the biosensor’s sensitivity and specificity. In recent years, optical fibers have shown significant advantages in areas such as lighting, laser gain media, and high-speed communications [19]. The phase, wavelength, light intensity, and other characteristic parameters of light waves transmitted in optical fibers change accordingly with changes in the external environment [20]. Based on several studies, it can be concluded that optical fibers have great application potential in herbicide detection [21,22,23].

Nanomaterials have been of substantial study interest in recent decades because of their unique physical, chemical and biological properties [24]. Gold nanoparticles (AuNPs) are regarded as the best immobilization materials by researchers due to their easy synthesis, controllable morphology and particle size, better biocompatibility, and higher extra-nuclear electron density [25]. The evanescent wave interacts with the free AuNPs when they are coated on the surface of the tapered region of optimized optical fiber [26]. The LSPR effect is highly sensitive to changes in the refractive index (RI), which improves the detection sensitivity of the sensor to target chemicals [27]. Molybdenum disulfide nanosheets (MoS_2_-NSs) have a large surface area, good optical and catalytic properties, relatively high electron mobility, and good biocompatibility, and they can be easily prepared and functionalized, which significantly improves the production efficiency and cuts down the production cost [28]. In addition, the large specific surface area of MoS_2_-NSs can supply numerous active sites to enhance the adsorption efficiency of target molecules, which is extremely favorable for improving the sensitivity of the sensor [29]. Zinc oxide nanowires (ZnO-NWs) have high strain-at-break properties and good biocompatibility, making them promising and noteworthy for nanoscale sensors [30]. Given the aforementioned benefits, MoS_2_-NSs and ZnO-NWs will be crucial to this experiment and have a lot of promise for use in fiber-based biosensors.

This study developed a plasmonic biosensor that integrates the LSPR phenomenon with ALP inhibition detection. The signal amplification capability of LSPR and the unique biological regulatory function of ALP inhibition complement and synergize with each other. The sensor was functionalized with MoS_2_-NSs and ZnO-NWs. The superior capabilities of MoS_2_-NSs and ZnO-NWs are utilized to boost the effective sensing area and detection signal for the extremely sensitive detection of 2,4-D.

## 2. Experimental Section

### 2.1. Materials

Single-mode fiber (SMF) (8.2 µm, 125 µm) purchased from EB-Link Technology Co., Ltd. (Shenzhen, China). The suppliers of sodium hydroxide (NaOH) and ALP were MackLin (Shanghai, China), and 2,4-D was purchased from Aladdin (Shanghai, China). Shanghai Dibai Biotechnology Co., Ltd. (Shanghai, China) was the supplier of (3-mercaptopropyl) trimethoxysilane (MPTMS). Acetic acid was purchased from the China Yantai Far East Fine Chemical Co., Ltd. (Yantai, China). The AuNP solution was synthesized from deionized water, tetrachloroauric acid (Merck, Darmstadt, Germany), and trisodium citrate. MoS_2_-NS powder purchased from Sigma-Aldrich (St. Louis, MO, USA) was used to prepare the MoS_2_-NS solution with N-methyl-2-pyrrolidone (NMP). ZnO-NWs were purchased from Beijing Beike New Materials Technology Co. Ltd (Beijing, China). They have a diameter of 100 nm and a length of 2 μm [31]. Owing to their large aspect ratio, they possess a relatively large specific surface area of over 35 m^2^/g. Structurally, the ZnO-NWs are hexagonal columnar single crystals, presenting a white foam-like appearance with distinct particles and uniform dispersion [32]. These physical properties endow them with special performances in fields such as catalysis and optoelectronics [33]. Glyphosate, atrazine, lindane, paraoxon, and phosphate anions purchased from Liaocheng Xinyue Biotechnology Co., Ltd. (Liaocheng, China) and were used as target analytes in the selectivity assessment.

### 2.2. Instruments and Measurements

In this experiment, a 3SAE Combiner Manufacturing System (CMS, Franklin, TN, USA) was employed to prepare multi-tapered fiber structures. The nanoparticles on the fiber surface were studied by the scanning electron microscope (SEM, Carl Zeiss microscope, Jena, Germany). The microstructure of the nanomaterials was analyzed using high-resolution transmission electron microscopy (HR-TEM, Talos L120C, Thermo-Fisher Scientific, Waltham, MA, USA) to determine the shape and size of the AuNPs and MoS_2_-NSs/ZnO-NWs. An UV-Vis spectrophotometer (Hitachi-3310, Tokyo, Japan) was used to obtain the absorption spectrum of the AuNPs. A tungsten–halogen light source (HL-2000 Shanghai, China) with a broad spectrum was used as the excitation light source. A spectrometer (USB2000+ Shanghai, China) was used to process and record the detected spectral data on a computer.

### 2.3. Sensing Mechanism of the Probe

Metal nanoparticles are coated on the sensor to generate a strong resonance peak via the LSPR resonance mechanism. From Equation (1), it can be observed that the shift of the LSPR resonance wavelength (∆λ) is highly dependent on the dielectric constant and the composition of the sensor surrounding medium [34].

(1) $$∆\lambda =m\times ∆n\left[1-\exp\left(-\frac{2t}{d_{p}}\right)\right]$$

In Equation (1), m represents the total RI reaction, Δn indicates the degree of RI variation in the probe surroundings, and t shows the efficiency of the adsorbed layer depth. Total internal reflection of light gives rise to an evanescent wave at the interface between two materials of different indices of refraction. As the propagation distance increases, the potential gradually decays. When the potential decays to 1⁄e, the depth ($d_{p}$) of penetration can be expressed by Equation (2) [35]:

(2) $$d_{p}=\frac{\lambda }{2\pi \sqrt{n_{cl}^{2}\sin^{2}\alpha -n_{co}^{2}}}$$

The symbols λ, α represent the wavelength and angle of incidence of a beam at the interface between the core and cladding. $n_{cl}$, $n_{co}$ represent the refractive indices of the cladding and core of the SMF, respectively.

### 2.4. Fabrication Mechanism of the Probe

**Figure 1:**
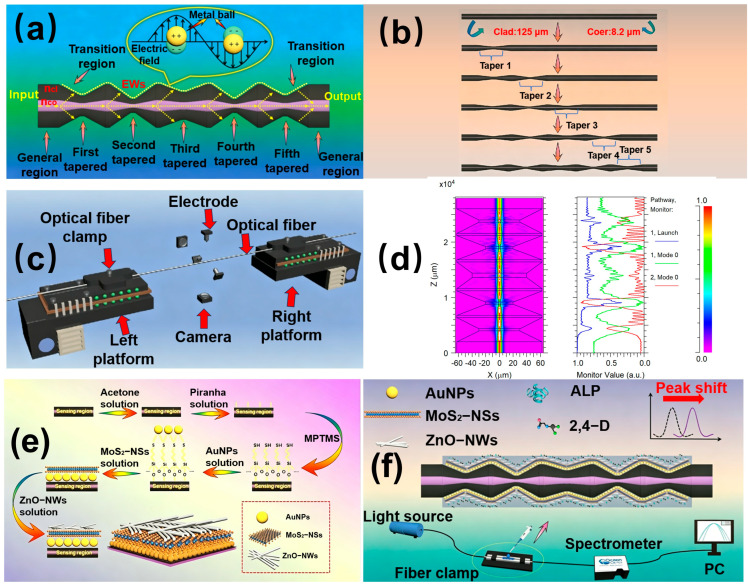
(**a**) Schematic representation of LSPR principle. It displays the process of interaction between light and matter. (**b**) Schematic diagram of fiber tapering process. (**c**) Schematic representation of the structure of the CMS device. (**d**) Simulation schematic diagram using the RSoft program. It depicts the light field distribution and mode propagation parameters in sensing region. (**e**) A flowchart of the nanoparticle coating process and describes the precise procedures for nanoparticle coating by chemical modification. (**f**) Schematic of the experimental setup for the detection of 2,4-D. If the RI in the taper region is changed, the spectrum shown on the computer will vary, too.

The biosensors designed for this experiment were fabricated using the CMS process. Before each use of the CMS machine, the CMS was calibrated and aligned using a silicon capillary tube. Simultaneously, the optical fiber needs to be pretreated by stripping the coating layer, ethanol rinsing, and dust-free paper wiping to eliminate the interference and influence of dust on this experiment. Pretreated fiber was tapered by setting Program 1 (waist diameter: 60 μm, total length of transition section: 3000 μm, total length of taper: 4000 μm) on the computer. Then, a second tapering was carried out behind the waist area of 60 μm by setting Program 2 (waist diameter: 40 μm, total length of the transition section: 4000 μm, total length of the taper: 5000 μm). Then, a third tapering was performed behind the 40 μm waist region by establishing Program 3 (waist diameter: 80 μm, total length of the transition section: 4000 μm, total length of the taper: 4800 μm). A fourth tapering was then performed in the 80 μm waist area with Program 2. Finally, a fifth taper was made behind the 40 μm waist region using Program 1. The tapered structural fiber required for this experiment was fabricated after 5 tapering steps as illustrated in Figure 1b. The light from the tapered structure fiber passes through the evanescent field on the fiber surface resonating with AuNPs as shown in Figure 1a, so as to produce the LSPR effect. Figure 1c shows the interior environment of the CMS. The CMS achieved the tapering of the material by heating and stretching the fiber.

### 2.5. Simulation of Fiber Sensor Structure

The RSoft simulation software (2020.03) was used in this experiment. The simulation model was first designed in RSoft CAD, and it was ensured that the model parameters were consistent with the multi-tapered structure fabricated in the experiment. The SMF had a core dimension of 8.2 μm and a cladding dimension of 125 μm. The cladding possessed an RI of 1.466, whereas the core had an RI of 1.467. The simulation results in Figure 1d show that light can be transmitted stably before entering the taper region. When light passes through the taper region, the modes in the core begin to couple with the cladding, leading to electron oscillations in the taper zone. The coupling between higher-order modes can be enhanced by establishing a multi-tapered structure. Part of the energy leaked from the coupling process is absorbed by the AuNP coating, thus enhancing the LSPR effect.

### 2.6. Synthesis of AuNPs, MoS_2_-NSs, and ZnO-NWs

First, 0.228 g of trisodium citrate was weighed into 2 mL of deionized water and mixed evenly with a rapid mixer. Then, 150 μL of chloroauric acid (100 mM) solution was added to 14.85 mL of deionized water and put on a magnetic stirrer. A magnetic stirrer was used to stir and heat the solution to boiling, and 1.8 mL of trisodium citrate (38.8 mM) was quickly added to the solution. The solution was then heated for 5 min and the heat was turned off. Finally, after 10 min of stirring, AuNPs were obtained. First, 30 mg of MoS_2_-NS powder was added to 10 mL of NMP solution, and it was placed in an ultrasonic machine to sonicate for 1 h. Then, it was centrifuged at 5000 rpm for 30 min, and the resulting suspension was used as MoS_2_-NSs. The ZnO-NWs were prepared by first weighing 10 mg of ZnO-NW powder and adding 10 mL of DI water and mixing them, and then placing them in an ultrasonic machine for one hour.

### 2.7. Immobilization of AuNPs, MoS_2_-NSs, and ZnO-NWs over Fiber Structure

AuNPs immobilized on the fiber surface first needed to be immersed for 12 h using 1% ethanol MPTMS solution (with 0.1 mL of MPTMS + 9.9 mL of ethanol); after immersion, the fiber taper area was cleaned using ethanol, and then dried by nitrogen gas and air-dried for half an hour; finally, the air-dried fiber was put into the prepared AuNPs solution for 48 h, and at the end of the immersion, it was again dried and air-dried with nitrogen gas for drying and air-drying treatment. For MoS_2_-NSs, they were first submerged in the prepared MoS_2_-NS dispersion liquid for 20 s. Next, the fiber was warmed in an oven at between 60 °C and 70 °C for 2 min. This process was repeated eight times. When coating with ZnO-NWs, the tapered fiber region was submerged in the ZnO-NW dispersion for ten min. Subsequently, the fiber was heated for 20 min at 60 °C to 70 °C in an oven. The specific nanomaterial immobilization process is shown in Figure 1e.

### 2.8. Experimental Setup for 2,4-D Detection

The first step was to use a fusion splicer to connect the optical fiber probe to the light source and then connect it to the spectrometer. When light from the light source passes over the surface of the probe, it excites the sensor to undergo LSPR. The hydrolysis reaction between ALP and sodium phenyl phosphate resulted in a shift in the significant wavelength detected by the spectrometer. Ultimately, 2,4-D was detected by observing the inhibitory effect of 2,4-D on the hydrolysis reaction between ALP and sodium phenyl phosphate. During the detection process, the 2,4-D concentrations were sequentially detected from low to high values. The sensor probe was cleaned with Tris(hydroxymethyl)aminomethane hydrochloride (Tris-HCL) buffer before each concentration detection, and a group of concentrations was detected several times to improve the accuracy of the experiment. Figure 1f shows the basic setup for the spectrum measurement experiment.

### 2.9. Preparation of 2,4-D Detection

Next, 1 mg of 2,4-D was added to a test tube along with 10 mL of Tris-HCL buffer, and the combination was thoroughly stirred to yield a 100 mg/L 2,4-D solution. Tris-HCL buffer was used for dilution of 2,4-D solutions of varied concentration. The experiment consisted of preparation of 2,4-D solutions at concentrations of 1, 2, 6, 8, and 10 mg/L.

## 3. Results and Discussion

### 3.1. Optimization of Optical Fiber Structure

**Figure 2:**
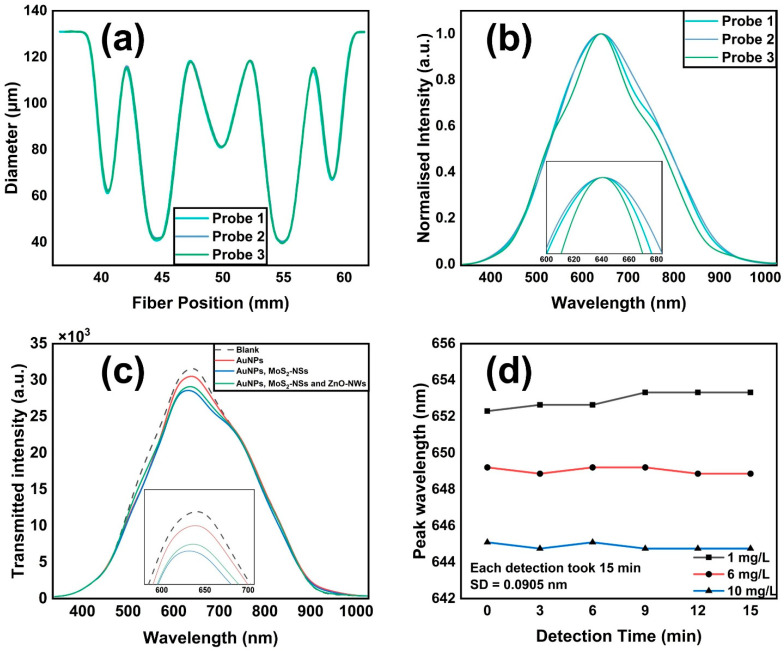
(**a**) Scanning image of the fiber structure diameter after scanning. The tapered portions of the three fibers are uniform well. (**b**) Normalized transmission intensity spectrum of the optical fiber exhibiting the spectral response characteristics and the baseline optical performance of the probe before functionalization. The inset is a locally enlarged view. (**c**) Plot of intensity of transmission vs functionalization showing that the intensity of transmission decreases with the coating of nanomaterials. The inset is a locally enlarged view. (**d**) Graph of the fiber optic detection time analysis revealing that the signal stabilizes 12 min after the probe encounters the analyte.

CMS machines help optimize optical fibers by creating uniformly high-temperature zones through the arc-discharge technique. Parameters such as electrode power, motor speed, air pressure threshold, excessive region length, and taper diameter were used to design the multi-tapered structure by continuously varying the CMS program. During the optimization of the multi-tapered structure, as the number of tapers increased, the sensing region gradually enlarged and the surface energy was enhanced, which in turn contributed to the continuous enhancement of the evanescent fields. However, when the number of tapers was more than five, the increase in the number of tapers had negligible influence on the cladding energy. Therefore, a five taper multi-taper construction was selected. The transmission performance of the fiber can be further enhanced by combining procedures with different parameters, such as 40 µm, 60 µm, and 80 µm. The dimensions of the three created fiber structures were measured using the CMS scan function, which shows good reproducibility of the fabricated fiber structures, reducing experimental errors, as shown in Figure 2a. The transmission spectra of the developed fiber-optic sensors are presented in Figure 2b. The peaks of the fibers were found to be overlapped and almost identical, showing good reproducibility of the produced fibers. In addition, the transmission powers of the fiber optic probes were examined before and after coating with different nanomaterials. As shown in Figure 2c, the transmission intensity decreased after nanomaterial functionalization because of the increase in surface roughness. This added more scattering centers during the propagation of light, resulting in part of the optical energy scattering in different directions, thereby decreasing the intensity of the light received at the transmission end. This also indirectly confirmed that the nanoparticles were successfully loaded on the surface of the fiber.

Stability is a crucial component in assessing the detection capacity of a sensor. To determine the detection time, a 15 min detection experiment was conducted using the probe for 2,4-D solutions with concentrations of 1, 6, and 10 mg/L. Initially, 500 µL of a 1 mg/L solution was placed near the sensor head, and the LSPR spectra of the transmitted intensity were recorded at 1 min intervals throughout the detection process. After 15 min, the sensing area was rinsed with Tris-HCL buffer and allowed to dry for 10 min at room temperature. Subsequently, the detection time was tested again using solutions of 6 mg/L and 10 mg/L concentrations. As shown in Figure 2d, the peak wavelengths obtained from each measurement were summarized and plotted against the detection time. The resonance wavelengths of the three different-concentration solutions all converged to a linear steady state after 12 min and remained stable for the next 3 min. Based on this, the data recording interval was set to 15 min for all concentrations of the solutions throughout the experiment.

### 3.2. Nanomaterial Characterization of the Fiber Probe

**Figure 3:**
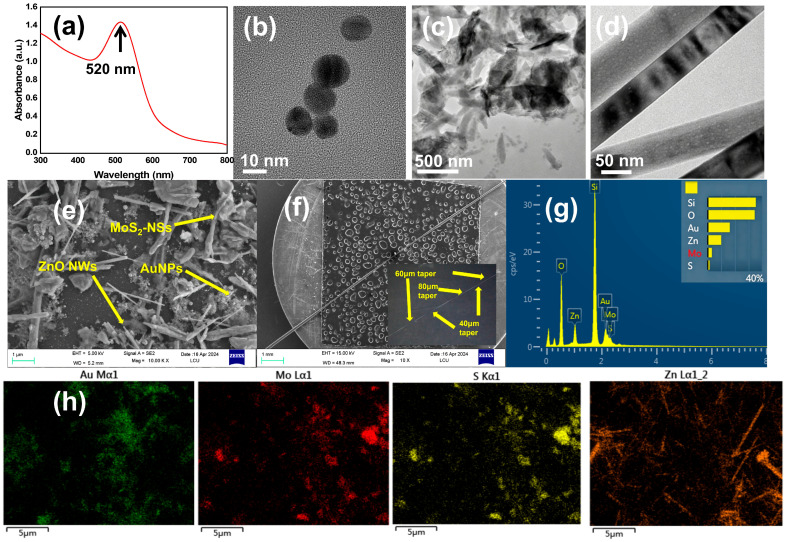
(**a**) UV–visible absorption spectra of AuNPs showed plasmon resonance activity at 520 nm. HR-TEM pictures showing the morphological and structural properties of (**b**) AuNPs, (**c**) MoS_2_-NSs, and (**d**) ZnO-NWs. (**e**) SEM image of the nanomaterials coated on the surface of the optical fiber, showing good coverage of the coating on the sensing area. (**f**) SEM picture of the tapered fiber structure. The inset is a locally enlarged view. (**g**) The SEM-EDS analysis of the product shown in Figure (**e**) validated the elemental makeup of the nanomaterials and their effective deposition on the fiber structure. (**h**) Distribution map of elements in the nanoparticles in Figure (**e**). The distribution of the nanoparticles on the optical fiber.

AuNPs, MoS_2_-NSs, and ZnO-NWs were characterized by HR-TEM to study the microstructure of the materials. The absorption spectrum of AuNPs (Figure 3a) was measured using a UV-Vis spectrophotometer and showed an absorption peak at 520 nm, suggesting the diameter of AuNPs to be about 10–15 nm [36]. The as-prepared AuNPs were characterized by HR-TEM and are displayed in Figure 3b. The AuNPs reveal a homogeneous spherical structure with a diameter of about 10–15 nm. The sheet-like layered structure of MoS_2_-NSs is shown in Figure 3c and the uniform cylindrical nanowire shape of ZnO-NWs is shown in Figure 3d. As shown in Figure 3e, AuNPs, MoS_2_-NSs, and ZnO-NWs are all attached to the fiber sensing area. The SEM picture of the tapered fiber structure is shown in Figure 3f. SEM-EDS analysis was carried out to determine the elemental composition of the cone region, as shown in Figure 3g, in which O, Zn, Au, Mo, and S elements were detected, further confirming the successful attachment of ZnO-NWs, AuNPs, and MoS_2_-NSs. Figure 3h further shows the distribution of the nanoparticle elements in Figure 3e.

### 3.3. Analytical Performance and Calibration for ALP and 2,4-D Detection

**Figure 4:**
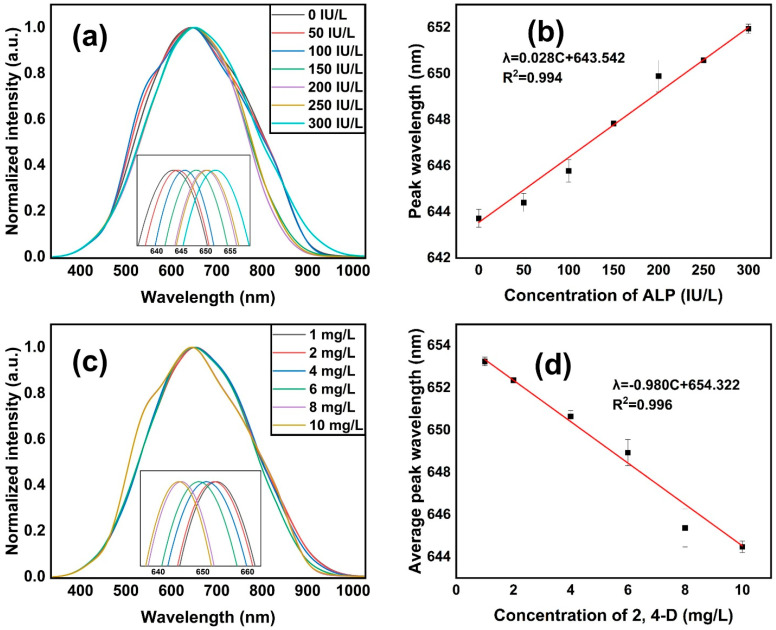
(**a**) Sensor spectrum for ALP detection with the red-shift of the wavelength as a function of the analyte concentration. The inset is a locally enlarged view. (**b**) Linear calibration graph of ALP detection with a good linear fit of the sensor response and analyte concentration. (**c**) The spectral detection of 2,4-D sensor exhibits blue-shift in wavelength owing to change in concentration of target analyte. The inset is a locally enlarged view. (**d**) Linear calibration graph for the detection of 2,4-D showing strong linear fitting between the sensor response and analyte concentration.

ALP is a metalloenzyme that contains zinc. The enzymatic activity requires three metal ions in the active site: two Zn^2+^ and one Mg^2+^ [37]. 2,4-D enters straight into the active site of ALP, hence inhibiting its catalytic activity. The inhibitor binds to the metal ions at the active site of the enzyme or occupies the substrate binding site, therefore preventing the enzyme from catalyzing substrate hydrolysis and inhibiting the signal output [38]. In this study, we measured the enzyme activity as International Units per liter (IU/L). IU/L is related to the enzyme’s catalytic ability in biological samples, which is closely related to the objectives of our study and allows us to assess the changes in enzyme activity under different conditions. One U of enzyme activity is defined as the amount of enzyme which will convert 1 μM of substrate per min per liter of solution under given circumstances [39]. The sensor designed in this experiment first detected ALP in a measurement range of 0–300 IU/L, and the concentrations were detected sequentially from low to high, and the development trend of the sensor results was observed with the increase of ALP concentration, and finally the most effective concentration of 300 IU/L was selected to detect the herbicide 2,4-D. The sensing spectrum results of ALP are shown in Figure 4a, which indicates that the peak wavelength shows a red-shift with the rise of ALP concentration. The sensing findings in the ALP measurement are linearly plotted in Figure 4b. The R^2^ is 0.994, indicating a high degree of linear fit.

This investigation is focused on inhibition impact of herbicide 2,4-D on hydrolysis reaction of disodium phenyl phosphate catalyzed by ALP. This inhibition can be measured to find the presence of pesticide residues. In this experiment, the ALP concentration was adjusted to 300 IU with a starting activity of 300 IU/L. The spectra were stable after 10 min. In the experiment, the 2,4-D solution was tested with *n* = 5 replicates. The detection concentration range of 2,4-D is 1–10 mg/L. Each concentration was evaluated for 15 min. When changing the concentration of 2,4-D, the sensor region was washed with Tris-HCl buffer to avoid experimental error. Tris-HCl buffer is also an activating buffer, which can improve the catalytic activity of ALP and create a suitable pH environment for ALP to work properly, which is the effect of Tris-buffer on ALP. The hydrolysis reaction of 2,4-D with ALP causes the RI of the optical fiber sensing area to change, which results in a change in the signal. The LSPR peak value is changed after being sent to the laptop by the spectrometer. Then, the data were sorted and analyzed. The detection findings of the 2,4-D sensing spectrum are shown in Figure 4c. We see that the peak wavelength shows a blue-shift with the rise of 2,4-D content. The linear connection graph of the 2,4-D sensing spectrum results is shown in Figure 4d. The results demonstrate that the peak wavelength has a negative correlation with varied amounts of 2,4-D, and R^2^ is 0.996, indicating a strong linear fitting. The limit of detection (LOD) is an important parameter in the field of sensor applications, and it can be expressed using Equation (3) [40].

(3) $$LOD=\frac{3.3\times SD}{Sensitivity}$$

Here, SD is the result obtained from 15 consecutive measurements of the Tris-HCl buffer solution by the sensor. The result is 0.0883 nm. Sensitivity refers to the slope of the linear equation of the sensor with a value of 0.980 nm/(mg/L). From this calculation, the LOD was calculated to be 0.29 mg/L. We developed sensors with four (60-40-80-40 µm) and six (60-40-80-60-40-60 µm) cone zones for detecting 2,4-D solutions at concentrations ranging from 1 to 10 mg/L. The detection findings are listed in Table 1. The sensor with five cone sections has the highest sensitivity. Another important metric in the realm of sensor applications is the Limit of Quantification (LOQ), which can be expressed using Equation (4) [41].

(4) $$LOQ=\frac{10\times SD}{Sensitivity}$$

From this calculation, the LOQ was determined to be 0.89 mg/L.

**Table 1:** Sensitivity of detection of 2,4-D with various numbers of taper sections.

Number of Tapered Sections	Sensitivity
Four	0.876 nm/(mg/L)
Five	0.980 nm/(mg/L)
Six	0.952 nm/(mg/L)

### 3.4. Reliability, Selectivity, and Environmental Stability of the Developed Biosensor

**Figure 5:**
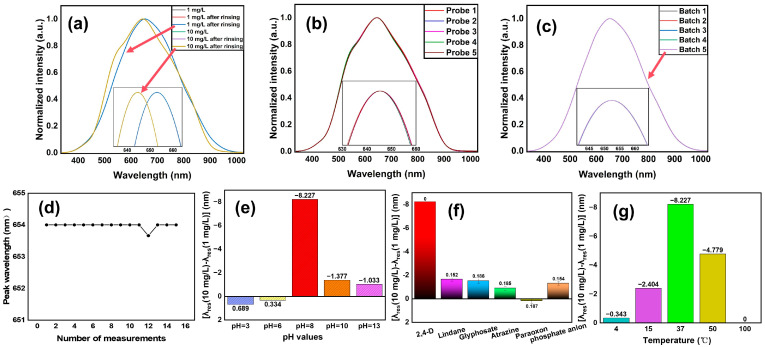
(**a**) Repeatability test: The sensor was tested three times at each concentration, confirming satisfactory repeatability. The inset is a locally enlarged view. (**b**) Reproducibility test: 5 sensors from the same batch are tested, showing the sensor has good reproducibility. The inset is a locally enlarged view. (**c**) Reproducibility test. The answers of the sensors from 5 separate batches were tested, showing good reproducibility of the sensor. The inset is a locally enlarged view. (**d**) Stability test: The biosensor exhibits good stability as shown in the performance change after a long time of operation. (**e**) pH value impact test: The influence of environmental pH conditions on enzyme activity was studied, and the sensor performed effectively at pH = 8. (**f**) Selectivity test: The good functionality of the sensor is shown by the selective recognition of the target analyte in the presence of possible interfering chemicals. (**g**) Temperature impact test: To study the influence of temperature fluctuation on sensor performance, the sensor works well at a temperature of 37 °C.

Repeatability tests were performed to check the consistency of many measurements with the same sensor probe at a given concentration, and to ensure the stable operation of the sensor probe. The experiment was performed at two concentrations (1 mg/L and 10 mg/L) with *n* = 3 replicates for each concentration. After the first signal recording, the probe was washed many times with Tris-HCl buffer prior to re-testing. The results are displayed in Figure 5a. The peak wavelengths are essentially the same. The 95% confidence interval of 1 mg/L in Table 2 is 652.6–653.6 nm and RSD is 0.030%. The 95% confidence interval for 10 mg/L is 644.3–644.8 nm, with a relative standard deviation (RSD) of 0.029%. This indicates a great repeatability of the probe. Reproducibility tests are performed in order to confirm that different probes provide the same response: the results in Figure 5b indicate that the peak wavelengths of the five optical fibers in the same batch were relatively stable when the concentration of 2,4-D was 10 mg/L. The 95% confidence interval is 645.3–645.6 nm with RSD of 0.022% (Table 2). In addition, five different optical fibers were tested with 2 mg/L of 2,4-D. The variance of the peak wavelength is modest, as shown in Figure 5c, confirming the good consistency of the probe. The 95% confidence interval is 652.2–652.5 nm (Table 2) and the RSD of 0.026% clearly demonstrates the outstanding reproducibility of the sensor.

Stability tests were used to ensure that sensor performance remains stable over long-term operation. The Tris-HCL solution was placed in the sensing region 15 times, and the spectra were recorded at 15 min. The sensing area was rinsed before each solution change, and, as shown in Figure 5d, the resonant peak wavelength of the sensor was consistently approximately 654 nm after 15 measurements. This result indicates that the sensor performance was stable, with a SD of 0.0883 nm. According to 2,4-D concentration, the observed results confirmed the good stability of the developed sensor.

The purpose of measuring the pH was to determine the ideal pH range for 2,4-D residue detection in experimental settings. First, solutions with different pH values were prepared by adding NaOH and acetic acid to obtain pH values of 3, 6, 8, 10, and 13. Second, solutions containing 1 mg/L and 10 mg/L of 2,4-D were prepared at pH values of 3, 6, 8, 10, and 13. The order of pH values was from low to high during the assay. Before each solution detection, the surface of the measurement probe was cleaned with Tris-HCL buffer. As shown in Figure 5e, the resonance offset is the largest and most obvious at pH = 8. Therefore, pH = 8 was selected as the optimal pH environment for the ALP in this experiment. However, the ALP enzyme protein denatures and causes the enzyme to be inactivated when the pH is more than 10 or less than 6.

The selectivity experiment evaluated whether the sensor probe could specifically recognize 2,4-D in the presence of multiple interferences. The sensor probe’s capacity to precisely detect 2,4-D in the presence of various interferents (glyphosate, atrazine, lindane, paraoxon, and phosphate anions) was evaluated. The tests were carried out using concentrations of interfering species within the same range (1–10 mg/L) as that used for the detection of 2,4-D, by measuring the peak shifts. This ensures the dependability of the experimental results. We used a sample size of *n* = 5 to measure the interferents. As shown in Figure 5f, only 2,4-D induced a pronounced wavelength shift of −8.227 nm, whereas the maximum shift caused by any of the five interferents was less than −1.8 nm. The experimental error was minor. The selected studies did not examine herbicides that are structurally comparable to auxins or chemicals that are known to inhibit phosphatases. However, the functional test results were good.

The aim of the temperature assay was to track the effect of temperature shifts on sensor performance and to identify the optimum operating temperature. Five gradients (4 °C, 15 °C, 37 °C, 50 °C, and 100 °C) were tested. As shown in Figure 5g, performance improved with increasing temperature in the low-temperature range and peaked at 37 °C. At higher temperatures, ALP lost activity, and the sensor could no longer effectively detect the enzyme-inhibition change induced by 2,4-D. Consequently, 37 °C was set as the optimal detection temperature.

### 3.5. Testing of Real Samples

This experiment was performed using actual samples to assess the sensing performance of the sensor in real-world applications. 2,4-D is widely used in the growth process of fruits and vegetables, and food safety is very important to people [42]; therefore, ordinary apple and Chinese cabbage were chosen as real samples to test the 2,4-D and verify the feasibility of the sensor. First, 1 g of apple and cabbage were individually juiced to obtain raw extracts. The juice was then transferred to centrifuge tubes and centrifuged at 5000 rpm for 10 min to separate the supernatant. Subsequently, the supernatant was filtered through a 0.22 µm membrane and diluted with Tris-HCl buffer at a 1:10 volume ratio to yield the final real sample solution ready for analysis. Using the prepared real sample solution, prepare 2,4-D solutions with concentrations of 1 mg/L and 10 mg/L. In the pH experiment, we tested the activity of the enzyme under different pH conditions and found that the enzyme could be in the most favorable catalytic environment by adjusting the pH to 8. By setting the pH to 8, the catalytic efficiency of the enzyme can be maximized, the inaccuracies due to fluctuations in enzyme activity can be reduced, and more accurate findings can be obtained.

Recovery was defined as the addition of a known amount of the substance to be measured to a sample, the subsequent determination of the amount of that substance in the sample, and the calculation of the ratio of the actual measured value to the amount added. This ratio can be used to assess the accuracy and reliability of 2,4-D measurements in actual samples. The RSD is used to measure the amount of change in the dataset relative to its mean value; the lower the RSD value, the higher the stability of the data. The calculation formula is shown in Equation (4). Table 3 demonstrates that the recovery of apple was in the range of 96.2–100.4% with an RSD of 3.1%. The recovery of cabbage was in the range of 83.8–108.8% with an RSD of 6.3%. Real samples contain many chemical components, including minerals, phenolic compounds, and polysaccharides. The components may interact with 2,4-D during the detection process and interfere with the detection signal. This led to a signal amplification with a recovery rate of 108.8%. In the apple environment, the practical LOQ was 1 mg/L, and in the Chinese cabbage environment, the practical LOQ was 0.98 mg/L. According to the results, it can be seen that the sensor sample has a high recovery rate and relatively low RSD and has a good detection in real samples.

(5) $$RSD=\frac{SD(a,\;b)}{Average(a,b)}$$

### 3.6. Performance Comparison of Proposed Sensors

The performance of the screening-level sensor produced in this experiment was compared with other known biosensors, shown in Table 4. The sensor can detect 2,4-D solutions at concentrations of 1–10 mg/L within 15 min. Although the LOD of this sensor is higher than that of certain sensors, it is still lower than that of some sensors [43,44]. However, it does not satisfy the regulatory maximum residue limit (MRL) of 30 µg/L of 2,4-D in food and water.

We developed a synergistic sensing technique based on AuNPs as the LSPR core to detect the inhibitory effect of 2,4-D on ALP-catalyzed hydrolysis of disodium phenyl phosphate. The strong localized electromagnetic fields of AuNPs amplify the interaction between the evanescent wave of the optical fiber and the functional thin-film layers, thus providing a basis for sensitive detection. MoS_2_-NSs possess the merits of a strong light absorption capacity, low imaginary part of dielectric constant, and big specific surface area, which can improve the sensitivity of refractive index sensing. ZnO-NWs act as signal amplifier to give the sensor a higher detection sensitivity. The synergistic effect of the AuNPs-MoS_2_-ZnO tri-layer structure facilitates the sensitive detection of 2,4-D. As demonstrated in Table 5, it is obvious that the sensitivity of the AuNPs-MoS_2_-ZnO tri-layer structure is higher than that of the single AuNPs and the two combinations of AuNPs with MoS_2_-NSs or ZnO-NWs. The synergistic effect between MoS_2_-NSs and ZnO-NWs can concurrently improve the interfacial localized electromagnetic field intensity and capture efficiency of target molecules, thus boosting the sensitivity of the sensor. This is an indication of the role of nanomaterials in increasing the sensitivity of sensors.

## 4. Conclusions

In this work, a fiber structure was designed with five taper regions to increase the evanescent field on the fiber surface. The sensor construction was verified by simulation and analysis using the RSoft software. The fiber surface was chemically modified and AuNPs were successfully deposited on fiber. When the AuNPs resonate with the incident light, the LSPR is excited. Then, MoS_2_-NSs and ZnO-NWs were coated on the fiber surface by physical adsorption to improve the detection ability of the sensor for 2,4-D. The first result is that the sensor is able to detect different concentrations of ALP. Next, the appropriate ALP level was found to determine 2,4-D concentration. Then, the trend and statistics of the inhibitory impact of 2,4-D on ALP hydrolysis were evaluated to qualitatively and quantitatively detect 2,4-D residues. The sensor can efficiently screen 2,4-D from 1 to 10 mg/L with a LOD of 0.29 mg/L. Compared to other sensors, this sensor has a comparatively low LOD, but still higher than that of SERS-based sensors. Further, the sensor has been verified for its reproducibility, repeatability, selectivity, pH adaptation, and real-sample detection capability, showing good reproducibility and specificity. In the future, we will focus on merging the sensing head with compact light sources and detectors to produce a portable detection system for speedy on-site screening. We will also use machine learning algorithms to deal with spectral data to improve the recognition accuracy for low-concentration 2,4-D.

## Figures and Tables

**Table 2:** Repeatability and reproducibility test results.

Experiment	Number of Repetitions	95% Confidence Interval (nm)	RSD (%)
Repeatability	N = 3	652.6–653.6 644.3–644.8	0.030 0.029
Same batch reproducibility	N = 5	645.3–645.6	0.022
Different batches reproducibility	N = 5	652.2–652.5	0.026

**Table 3:** Test results and recoveries of 2,4-D in real samples.

Samples	Recovery (%)	RSD (%)
Apple	96.2–100.4	3.1
Chinese cabbage	83.8–108.8	6.3

**Table 4:** Comparison of performance: existing 2,4-D sensors vs. proposed sensor.

Mechanism	Materials Used	Linear Range (mg/L)	Sensitivity	LOD (mg/L)	Ref.
Fluorescence	n.r. ^a^	3.3 × 10^−3^–0.304	n.r. ^a^	0.4	[45]
Electrochemistry	MWCNTs ^b^	2.1–110	0.84% inhibition/(μg/L)	1	[15]
SERS ^c^	AgNPs	2.21 × 10^−5^–2.21	0.001 Arbitr.Units/(mg/L)	0.08	[46]
HPLC-DAD	n.r. ^a^	10–28	n.r. ^a^	0.53	[47]
LSPR	AuNPs/MoS_2_-NSs/ZnO-NWs	1–10	0.980 nm/(mg/L)	0.29	This work

^a^ not reported; ^b^ Multi-walled carbon nanotubes; ^c^ Surface-enhanced Raman Scattering.

**Table 5:** Comparative study of proposed sensors.

Sensor Probe	Detection Range (mg/L)	Sensitivity (nm/(mg/L))	LOD (mg/L)
AuNPs-coated probe	1–10	0.472	0.62
AuNPs/MoS_2_-NSs-coated probe	1–10	0.678	0.43
AuNPs/ZnO-NWs-coated probe	1–10	0.559	0.52
AuNPs/MoS_2_-NSs/ZnO-NWs	1–10	0.996	0.29

## Data Availability

The datasets utilized and/or analyzed in this study can be obtained from the corresponding author upon reasonable request.
